# Supplementary material for: Patterns of Intron Gain and Loss in Fungi
Source: PLoS Biol. 2004 Nov 30;2(12):e422. doi: 10.1371/journal.pbio.0020422 (PMC532390; doi:10.1371/journal.pbio.0020422)
Supplement: Table S1 — Also available at http://genes.mit.edu/NielsenEtAl/. (4.3 MB ZIP). [file pbio.0020422.st001.zip › NielsenEtAl/html/849.html]

AN5447.1.NCU06803.1.MG02378.1.FG01572.1


```
 CLUSTAL W (1.82) Multiple Sequence Alignments - Introns Inserted


Sequence 1: NCU06803.1	520 aa
Sequence 2: MG02378.1	517 aa
Sequence 3: FG01572.1	568 aa
Sequence 4: AN5447.1	515 aa
Alignment Length: 574 aa
Number Identitical Residues: 306 aa
Alignment Score (without introns) 14328


MG02378.1 	--------------------------------------------MVHLTSIPSHEEADD-
NCU06803.1	--------------------------------------------MVHLTIIPKEDEIRDG
FG01572.1 	MSLAQHSESASPVPIIETDDQGNPIKSSNGYFPSKGKSSTLVDNMAHLSVVGGAHRGDDS
AN5447.1  	--------------------------------------------MVHLASIKKDDDFEP-
          	                                            *.**: :   .     

MG02378.1 	-------KIVSGVKKVHLQLANDEDKFTTSVYGSRFAIDDLPKHEMAENEMPKEVAYRMI
NCU06803.1	----LDIPLTGALKAVHLQLANDEDRFTTSVYGSKFAAADLPRHEMPDEEMPKEVAYRMI
FG01572.1 	DAASVRSTSSRASRRPEMKLASYQDEYTTSVYGSRFAGMDLPRHHMPENEMPRDIAYRMI
AN5447.1  	--------VVNRVDSIKLDTIE-EDDYSATVYGTRFATQQLPHAEMPDREMPREVAYRMI
          	                .:.  . :* ::::***::**  :**: .*.:.***:::*****

MG02378.1 	KDELSLDGNPMLN2LASFVTTYM0EEEAEKLMADSFSKNFIDYEEYPQSADIQNRCVAMI
NCU06803.1	KDELSLDGNPLLN2LASFVTTYM~EEEAEKLMTESLPKNFIDYEEYPQTADIQNRCVSMI
FG01572.1 	KDDLSLDNNPMLN2LASFVTTYM0EDEAEKLMAESFSKNFIDYEEYPQSADIQNRCVNMI
AN5447.1  	KDELSLDGNPMLN2LASFVTTYM0EDEAEKLMAESFSKNFIDYEEYPQSAEIQNRCVNMI
          	**:****.**:** ********* *:******::*:.***********:*:****** **

MG02378.1 	GRLFNAPVGASEGVGAVGTSCVGSSEAIMLAVLAMKKRWKNKRLAEGKSVDKPNLIMSSA
NCU06803.1	GRLFNAPVKDAEASSAVGTSSVGSSEAIMLGVLAMKKRWKNKRIAEGKPADKPNLIMSSA
FG01572.1 	GDLFHAPPG-----SSVGTSTVGSSEAIMLGVLAMKRRWKNRRIAEGKSTEHPNIVMSSA
AN5447.1  	ARLFNAPTDS-DTDHPMGTSTVGSSEAIMLGTLAMKKRWQNKRKAEGKDYSRPNIVMNSA
          	. **:**    .   .:*** *********..****:**:*:* ****  .:**::*.**

MG02378.1 	VQVCWEKATRYFEVEEKLVYCSPDRYVIDPKETVDLVDENTIGICVILGTTYTGEYEDVR
NCU06803.1	VQVCWEKATRYFEVEEKFVYCTPDRYVIDPKETVDLVDENTIGICCILGTTYTGEYEDVK
FG01572.1 	VQVCWEKATRYFEIDEKLVYCTPDRFVMDPEQAVDLCDENTIGMCAILGTTYTGEYEDIK
AN5447.1  	VQVCWEKAARYFDVEERYVYCTEERYVIDPQQAVDLVDENTIGICAILGTTYTGEYEDVK
          	********:***:::*: ***: :*:*:**:::*** ******:* ************::

MG02378.1 	AVNDLLNERGLETPIHVDAASGGFVAPFVVPDLEWDFRCDRVVSINVSGHKYGLVYPGVG
NCU06803.1	AVNDLLVERGLDTPIHVDAASGGFVAPFVVPDLEWDFRLKNVVSINVSGHKYGLVYPGVG
FG01572.1 	AINDLLVERNLDVPIHVDAASGGFVAPFVVPDLEWDFRCEKVVSINVSGHKYGLVYPGVG
AN5447.1  	AINDLLVERGLDCPIHVDAASGGFVAPFIHPTLQWDFRLEKVVSINVSGHKYGLVYPGVG
          	*:**** **.*: ***************: * *:**** ..*******************

MG02378.1 	WVVWRSAEFLPQELVFNINYLGADQASFTLNFSKGASQVIGQYYQLIRLGKHGYRAIMSN
NCU06803.1	WVVWRSAEYLPQELVFNINYLGADQASFTLNFSKGASQVIGQYYQLIRLGKHGYRAIMSN
FG01572.1 	WVIWRSPEFLPQELVFNINYLGADQSSFTLNFSKGASQVIGQYYQLIRLGKHGYRAIMSN
AN5447.1  	WVVWRSPEFLPKELIFNINYLGAEQASFTLNFSKGASHVIGQYYQMIRLGKRGYRSVMVN
          	**:***.*:**:**:********:*:***********:*******:*****:***::* *

MG02378.1 	LTRTADYLSDSLEALGFGIMSKKSGEGLPLVAFRLT-----PDEDRIYDEFAIAHQLRVR
NCU06803.1	LTRTADYLAESLAALGFIIMSQKSGQGLPLVAFRLK-----EDPDRTYDEFALAHQLRVR
FG01572.1 	LTRTADYLTETLENMGFVIMSERSGAGLPLVAFRFKTVDEGGDPERHYDEFALAHHLRSR
AN5447.1  	ITRIADYLADQLEQLGFIIMSQRRGRGLPLVAFRLP----ADRADETFDEFAIAHQLRER
          	:** ****:: *  :** ***:: * ********:     .   :. :****:**:** *

MG02378.1 	GWVVPAYTMAPHTENLKMLRVVVREDFTRNRCDALIADVKLSLELLNQMDKKELERHQD~
NCU06803.1	GWIVPAYTMAPKTEGLKMLRIVVREDFSRNRCDGLISDIRSCQGILEQMDKETVKKQQE~
FG01572.1 	GWVVPAYTMAPNSG-VKMLRVVVREDFTKSRCDQLICDVKLCHGLLKETDQESIKKREE~
AN5447.1  	GWIVPAYTMAPHSNNLKLMRVVVREDFSMSRCDQLLSDIKLALKSLREMDQAMLERYTQ2
          	**:********:: .:*::*:******: .*** *:.*:: .   *.: *:  :::  : 

MG02378.1 	VIHKHGTHSGKASHNHPRFR0KEKHSLQGKHGKTHAVC
NCU06803.1	FIHKHHVVSGKASHNHPKYH0-EKHSLQGKTGKTHSIC
FG01572.1 	YIKNHIVTMGRGKHSHPVYK0DEQHSLQGKHGKTHAIC
AN5447.1  	HVRSHTTKSHRAKHTHPHYK~NETHSLQGRTGKTHGVC
          	 ::.* .   :..*.** :: .* *****: ****.:*
```
